# Coronary Artery Hematoma Treated with Fenestration Using a Novel NSE Alpha® Scoring Balloon

**DOI:** 10.1155/2017/8189530

**Published:** 2017-10-02

**Authors:** Naohiro Funayama, Takao Konishi, Tadashi Yamamoto, Daisuke Hotta

**Affiliations:** Hokkaido Cardiovascular Hospital, Hokkaido, Japan

## Abstract

The optimal management of coronary intramural hematoma has not been defined. We described a case in which coronary occlusion developed due to an intramural hematoma after percutaneous coronary intervention for mid left circumflex artery (LCX). Intravascular ultrasound (IVUS) demonstrated the progression of the intramural hematoma and a totally compressed true lumen. Our approach was based on fenestration with a scoring balloon (NSE Alpha, Goodman, Japan), which allowed the deployment of an additional stent to be avoided. In conclusion, this management can be effectively and safely performed.

## 1. Introduction

Intramural hematoma after percutaneous coronary intervention (PCI) occurs in up to 6.7% of cases [1]. Intramural hematoma is defined as an accumulation of blood within the medial space displacing the internal elastic membrane inward and the external elastic membrane outward, with or without identifiable entry or exit points [1]. Further expansion of this mural hematoma may lead to compression of the true lumen of the coronary artery and result in myocardial ischemia or infarction. Bailout management of the hematoma through the use of stenting or fenestration with a cutting or scoring balloon, or chronic total occlusion-dedicated guidewire, has been reported; however, no optimal treatment approach has yet been established. We present an interesting case of a patient who was successfully treated by coronary artery fenestration with scoring balloon angioplasty.

## 2. Case Presentation

**Figure 1:**
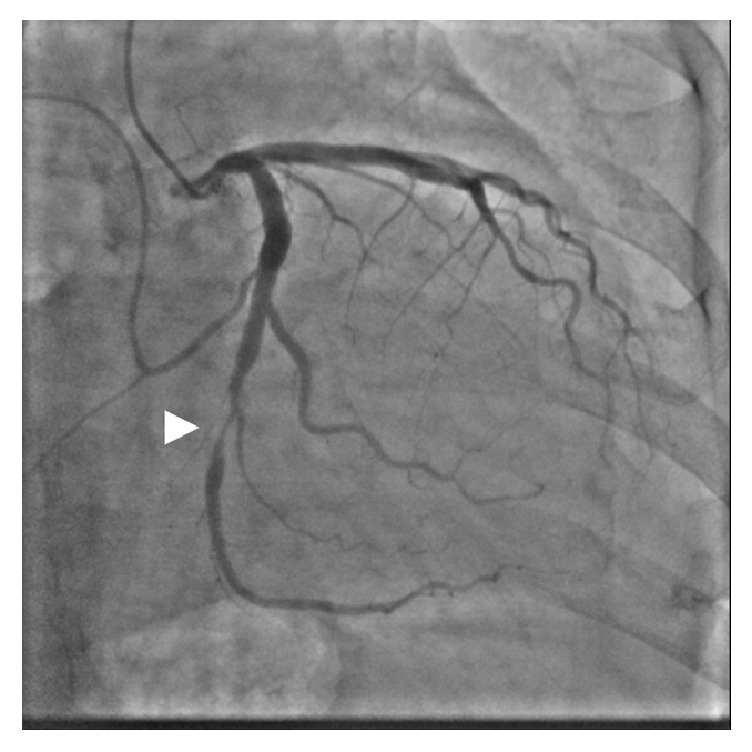
Baseline coronary angiography showed severe luminal stenosis of the mid left circumflex artery (head arrow).

**Figure 2:**
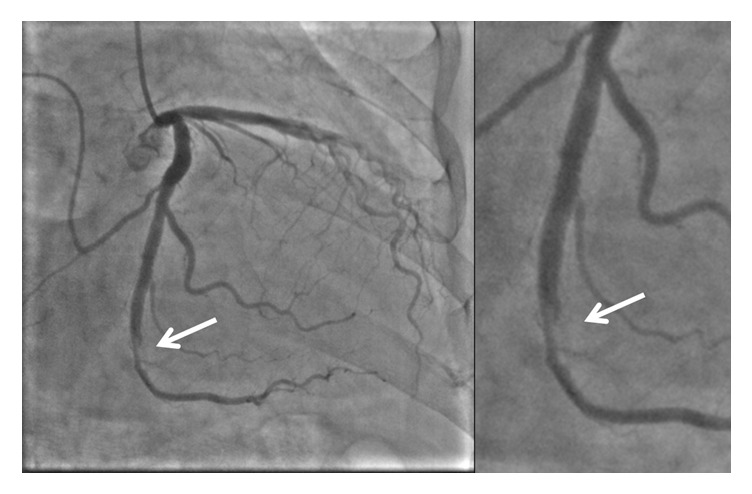
Angiography after stenting showed mild luminal narrowing with a dissection at the distal left circumflex artery (arrow).

An 84-year-old man with a history of hypertension and dyslipidemia was admitted to our hospital for the evaluation of chest pain. A twelve-lead electrocardiogram at rest demonstrated a normal sinus rhythm and no significant ST segment change. Coronary angiography revealed severe luminal stenosis of the mid left circumflex artery (Figure 1). We decided to perform elective PCI for the mid-LCX. We started PCI with a 5 Fr Heartrail II IL4 guide catheter and 0.014-inch Runthrough hypercoat guidewire (Terumo, Japan). The lesion was predilated twice with a semicompliant balloon (3.5 × 15 mm); however, distal slippage of the balloon occurred the first time. A 3.5 × 18 mm sirolimus-eluting stent was implanted at the target lesion. Although the final angiography showed residual mild luminal narrowing with a dissection at the distal LCX, there was no limitation of blood flow, so we concluded the procedure (Figure 2).

**Figure 3:**
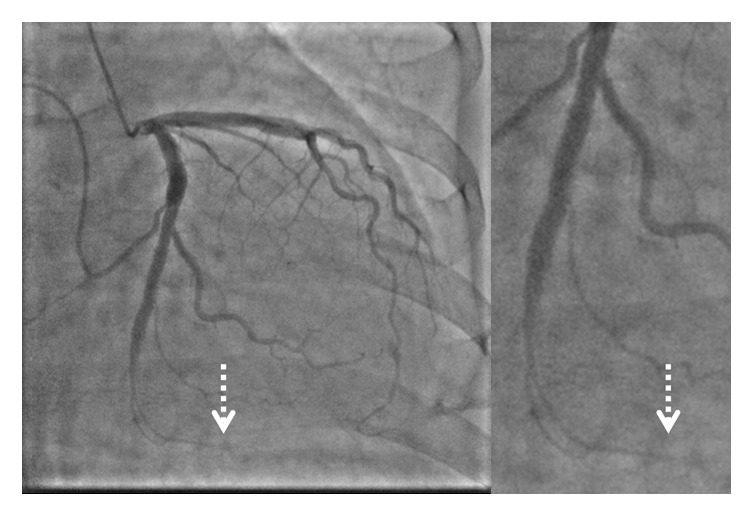
Emergent angiography revealed occlusion of the distal segment of the left circumflex artery (dotted arrow).

**Figure 4:**
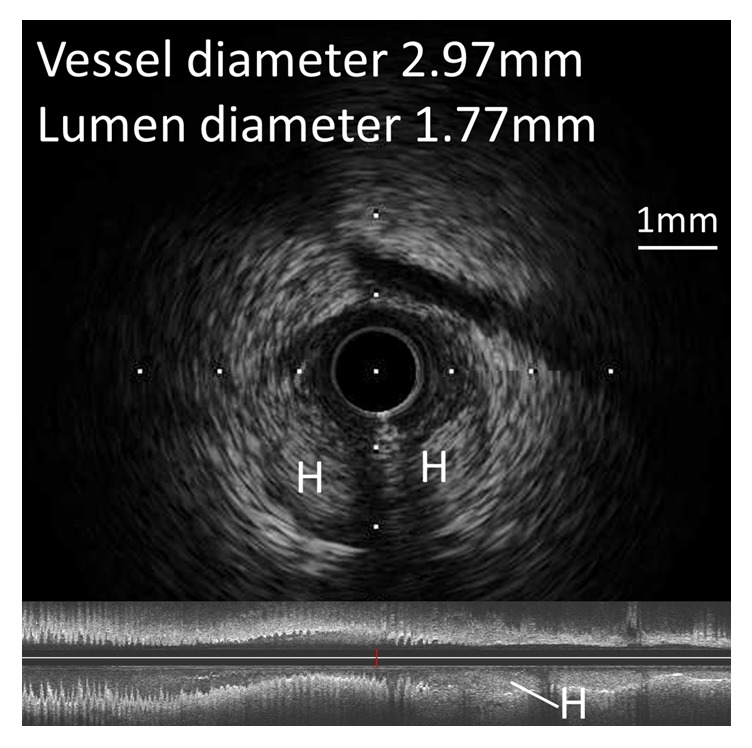
IVUS revealed progression of the intramural hematoma (H) and a totally compressed true lumen at the distal left circumflex artery. An intramural hematoma appears as a homogeneous, hyperechoic, crescent-shaped area.

**Figure 5:**
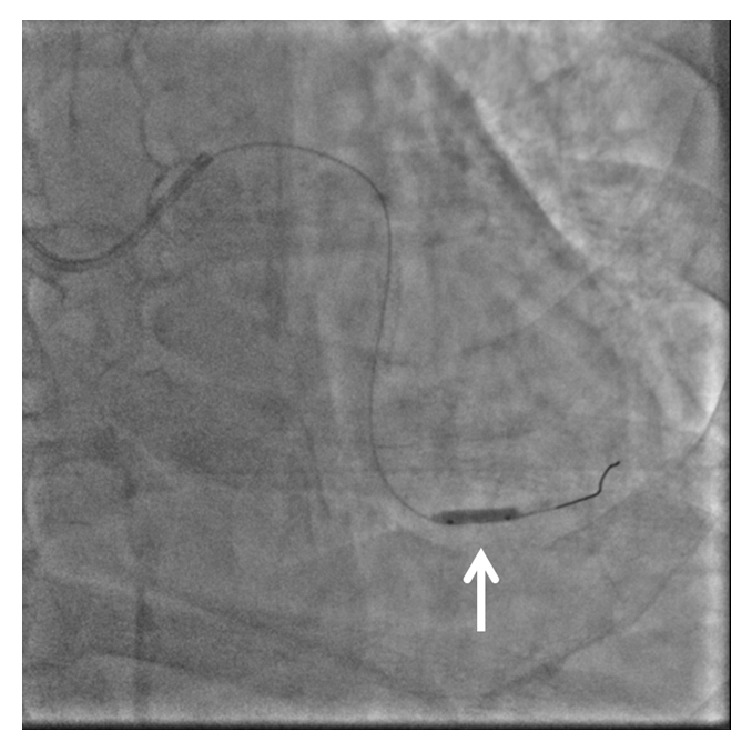
A novel scoring balloon (arrow: NSE Alpha 2.5 × 13 mm, Goodman, Japan) was dilated in the far distal LCX.

**Figure 6:**
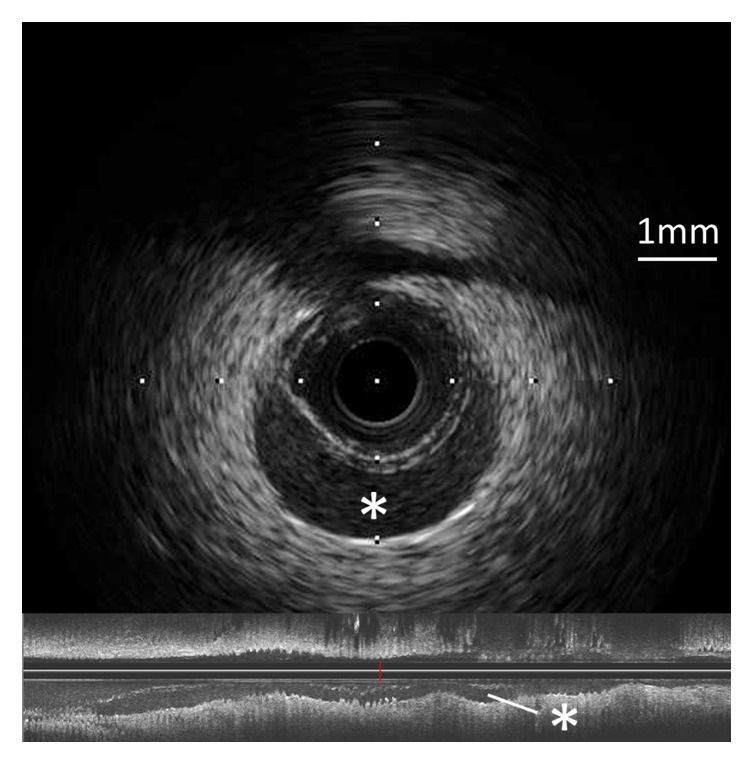
IVUS after fenestration with a scoring balloon showed the resolution of the hyperechoic area within the hematoma space (*∗*).

**Figure 7:**
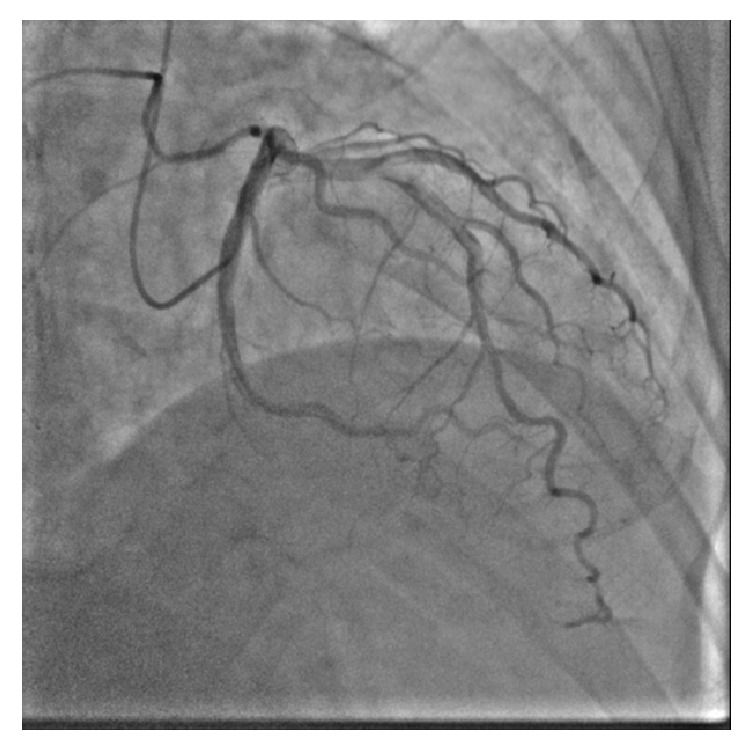
Angiography after fenestration showed Thrombolysis in Myocardial Infarction 3 (TIMI-3) flow.

Two hours later, the patient experienced chest pain and his electrocardiogram showed ST segment elevation in leads, II, III, aVF, and V5-6. Emergent angiography revealed occlusion of the distal segment of the LCX (Figure 3). We began the procedure with a 6 Fr back-up guiding catheter and inserted a 0.014-SION blue guidewire (ASAHI INTECC, Japan) into the distal LCX. Intravascular ultrasound (IVUS) revealed progression of intramural hematoma and a totally compressed true lumen at the distal LCX, a little apart from the distal edge of the stent (Figure 4). We suspected that predilation at the time of the first PCI might have damaged the intima and caused a dissection, leading to the intramural hematoma. It appeared difficult to achieve adequate coverage with stenting as the distal vessel was small and the end of the intramural hematoma could not be detected. We decided to treat the lesion by creating a fenestration between the true lumen and the hematoma and thereby decompressing the hematoma. A novel scoring balloon (NSE Alpha 2.5 × 13 mm, Goodman, Japan) was dilated 5 times at 6 atmospheres in the far distal LCX (Figure 5). IVUS images demonstrated the resolution of the hyperechoic area within the hematoma space; therefore, we considered a reentry to have been created (Figure 6). Angiography showed TIMI-3 flow with a long dissection and the resolution of luminal narrowing (Figure 7). After the procedure, the patient's complaints and ECG changes were resolved. Cardiac enzymes were increased (creatine kinase (CK) 819). He was discharged in a very good condition and his clinical outcomes were excellent at 1 month after intervention.

## 3. Discussion

Intramural hematoma after percutaneous coronary intervention (PCI) occurs in up to 6.7% of cases. Intravascular ultrasound (IVUS) has also identified intramural hematoma after 3.2% of DES implantation procedures [2]. Intramural hematoma is defined as an accumulation of blood within the medial space displacing the internal elastic membrane inward and the external elastic membrane outward, with or without identifiable entry or exit points [1].

The clinical presentations of intramural hematoma depend on the degree of pressure-driven enlargement of the hematoma space, with progressive true lumen compression resulting in myocardial ischemia or infarction [3].

It may be difficult to visualize the dissection and hematoma by coronary angiography [4], and IVUS and optical coherence tomography (OCT) can be valuable in establishing the correct diagnosis and in planning the management procedure. These images can provide important information on the extension of the hematoma and the decompression of the intramural hematoma after PCI. On IVUS images, an intramural hematoma typically appears as a homogeneous, hyperechoic, crescent-shaped area. The echogenicity of blood depends on the flow rate, red cell aggregation, and fibrin content [1]. In this case, the IVUS initially revealed images typical of an intramural hematoma as described above. However, after dilation with the scoring balloon, the IVUS showed that the hyperechoic area within the hematoma space had been resolved. We considered a reentry to have been created and blood flow in false lumen resumed, with resolution of red blood cell stagnation. OCT with high resolution imaging may enable a more accurate assessment and characterization of intramural hematomas. However, it is necessary to pay close attention not to further extend the hematoma by the injection of contrast medium when using OCT [3].

**Figure 8:**
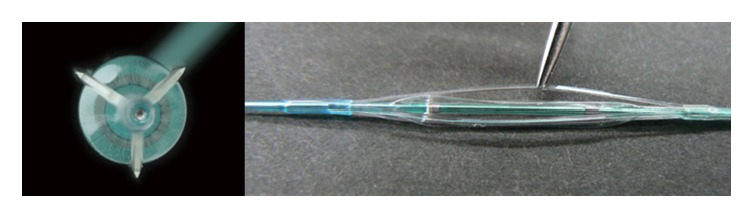
The NSE Alpha has 3 longitudinal nylon triangle elements mounted on the balloon surface to prevent balloon slippage during inflation.

Although there are several management approaches for intramural hematoma, the optimal treatment approach remains controversial. It has already been reported that stenting to restore distal flow in a narrowed vessel is an effective management approach. However, there are some concerns that stenting may cause longitudinal hematoma extension and is associated with an increased rate of restenosis and stent thrombosis [3, 5]. Some studies have reported good results for fenestration using cutting balloons [6], scoring balloons [7], and stiff guidewires [8]. Creating a fenestration between the true lumen and the hematoma leads to a reduction in pressure in the intramural hematoma. In this case, we decided to use a novel scoring balloon to make an “intentional reentry.” The NSE Alpha has 3 longitudinal nylon elements mounted on the balloon surface to prevent balloon slippage during inflation (Figure 8). The triangular elements of the NSE Alpha concentrate a greater degree of pressure on the vessel wall and although the concepts underlying a number of scoring balloon catheters appear to be similar, it seems that NSE Alpha is more effective in lesion dilation than the other scoring balloons due to the higher and sharp elements. Further, the concept of lesion dilation using the NSE Alpha is similar to that of a cutting balloon. However, a cutting balloon is more effective, although it has a limited crossability of the lesion due to the sharp metal microtome blades and risk of coronary rupture. Therefore, it remains possible that dilation with the NSE Alpha affords an effective and safety approach to fenestration. Accurate measurement of the hematoma diameter is necessary to determine the optimal balloon size to enhance the efficacy of the balloon and prevent further injury. In this case, the NSE Alpha diameter was determined based on the IVUS assessment, and we chose an NSE Alpha 2.5 mm, which is larger than the true lumen diameter but smaller than the vessel wall diameter. We also inflated the balloon in a section of the lesion shown to have less plaque by IVUS assessment in order to easily demonstrate the scoring efficacy.

## 4. Conclusion

Fenestration with an optimal scoring balloon based on IVUS measurement is an effective approach to the management of intramural hematoma and eliminates the need for the unnecessary deployment of a stent.
